# Adaptive genomic plasticity in large-genome, broad-host–range vibrio phages

**DOI:** 10.1093/ismejo/wraf063

**Published:** 2025-04-04

**Authors:** Charles Bernard, Yannick Labreuche, Carine Diarra, Pauline Daszkowski, Karine Cahier, David Goudenège, Martin G Lamarche, Gregory B Whitfield, Manon Lang, Jeffrey Valencia, Justine Groseille, Damien Piel, Yan-Jiun Lee, Peter Weigele, Yves V Brun, Eduardo P C Rocha, Frédérique Le Roux

**Affiliations:** Institut Pasteur, Université de Paris, Centre nationale de la recherche scientifique (CNRS), Unité mixte de recherche (UMR) 3525, Microbial Evolutionary Genomics, 28 rue du Dr Roux, 75015 Paris, France; Institut Français de recherche pour l'exploitation de la mer (IFREMER), Unité Physiologie Fonctionnelle des Organismes Marins, ZI de la Pointe du Diable, CS 10070, F-29280 Plouzané, France; Sorbonne Universités, CNRS, UMR 8227, Integrative Biology of Marine Models, Station Biologique de Roscoff, 29688 Roscoff, France; UMR 5244, Interactions hôtes-pathogènes-environnements (IHPE), Université de Montpellier, CNRS, IFREMER, Université de Perpignan via Domitia, Montpellier F-34090, France; Département de Microbiologie, Infectiologie et Immunologie, Université de Montréal, 2900, boul. Édouard-Montpetit, QC H3T 1J4 Montréal, Canada; Institut Français de recherche pour l'exploitation de la mer (IFREMER), Unité Physiologie Fonctionnelle des Organismes Marins, ZI de la Pointe du Diable, CS 10070, F-29280 Plouzané, France; Sorbonne Universités, CNRS, UMR 8227, Integrative Biology of Marine Models, Station Biologique de Roscoff, 29688 Roscoff, France; Institut Français de recherche pour l'exploitation de la mer (IFREMER), Unité Physiologie Fonctionnelle des Organismes Marins, ZI de la Pointe du Diable, CS 10070, F-29280 Plouzané, France; Sorbonne Universités, CNRS, UMR 8227, Integrative Biology of Marine Models, Station Biologique de Roscoff, 29688 Roscoff, France; Institut Français de recherche pour l'exploitation de la mer (IFREMER), Unité Physiologie Fonctionnelle des Organismes Marins, ZI de la Pointe du Diable, CS 10070, F-29280 Plouzané, France; Sorbonne Universités, CNRS, UMR 8227, Integrative Biology of Marine Models, Station Biologique de Roscoff, 29688 Roscoff, France; Département de Microbiologie, Infectiologie et Immunologie, Université de Montréal, 2900, boul. Édouard-Montpetit, QC H3T 1J4 Montréal, Canada; Département de Microbiologie, Infectiologie et Immunologie, Université de Montréal, 2900, boul. Édouard-Montpetit, QC H3T 1J4 Montréal, Canada; Département de Microbiologie, Infectiologie et Immunologie, Université de Montréal, 2900, boul. Édouard-Montpetit, QC H3T 1J4 Montréal, Canada; Département de Microbiologie, Infectiologie et Immunologie, Université de Montréal, 2900, boul. Édouard-Montpetit, QC H3T 1J4 Montréal, Canada; Sorbonne Universités, CNRS, UMR 8227, Integrative Biology of Marine Models, Station Biologique de Roscoff, 29688 Roscoff, France; Sorbonne Universités, CNRS, UMR 8227, Integrative Biology of Marine Models, Station Biologique de Roscoff, 29688 Roscoff, France; Biochemistry and Molecular Division, New England Biolabs, 240 County Road Ipswich, MA 01938, United States; Biochemistry and Molecular Division, New England Biolabs, 240 County Road Ipswich, MA 01938, United States; Département de Microbiologie, Infectiologie et Immunologie, Université de Montréal, 2900, boul. Édouard-Montpetit, QC H3T 1J4 Montréal, Canada; Institut Pasteur, Université de Paris, Centre nationale de la recherche scientifique (CNRS), Unité mixte de recherche (UMR) 3525, Microbial Evolutionary Genomics, 28 rue du Dr Roux, 75015 Paris, France; Institut Français de recherche pour l'exploitation de la mer (IFREMER), Unité Physiologie Fonctionnelle des Organismes Marins, ZI de la Pointe du Diable, CS 10070, F-29280 Plouzané, France; Sorbonne Universités, CNRS, UMR 8227, Integrative Biology of Marine Models, Station Biologique de Roscoff, 29688 Roscoff, France; Département de Microbiologie, Infectiologie et Immunologie, Université de Montréal, 2900, boul. Édouard-Montpetit, QC H3T 1J4 Montréal, Canada

**Keywords:** Schizotequatroviruses, broad host range, large genome, recombination, vibrio, coevolution, trade-off

## Abstract

The host range of a bacteriophage—the diversity of hosts it can infect—is central to understanding phage ecology and applications. Whereas most well-characterized phages have narrow host ranges, broad-host–range phages represent an intriguing component of marine ecosystems. The genetic and evolutionary mechanisms driving their generalism remain poorly understood. In this study, we analyzed Schizotequatroviruses and their *Vibrio crassostreae* hosts, collected from an oyster farm. Schizotequatroviruses exhibit broad host ranges, large genomes (~252 kbp) encoding 26 transfer ribonucleic acids, and conserved genomic organization interspersed with recombination hotspots. These recombination events, particularly in regions encoding receptor-binding proteins and antidefense systems, highlight their adaptability to host resistance. Some lineages demonstrated the ability of receptor-switching between OmpK and LamB. Despite their broad host range, Schizotequatroviruses were rare in the environment. Their scarcity could not be attributed to burst size, which was comparable to other phages *in vitro*, but may result from ecological constraints or fitness trade-offs, such as their preference for targeting generalist vibrios in seawater rather than the patho-phylotypes selected in oyster farms. Our findings clarify the genetic and ecological variables shaping Schizotequatrovirus generalism and provide a foundation for future phage applications in aquaculture and beyond.

## Introduction

Bacteriophages (or phages)—viruses that infect bacteria—are attracting renewed attention due to their ecological significance, impact on human health, and potential applications [1–3]. Effective therapeutic use of phages requires a host range that encompasses the genetic diversity of bacterial pathogens [4]. Host range is primarily determined by the phage’s ability to bind specific bacterial receptors, with variations in receptor-binding proteins broadening or narrowing this range [5, 6]. Bacterial defense mechanisms (e.g. [7]) also influence phage infection, and phages evolve rapidly to counter these defenses, with environmental context shaping the co-evolutionary dynamics between phages and bacteria [8]. Phages exhibit a wide range of host specificities, from those with highly restricted host ranges to others with broad infectivity, though most well-characterized model phages tend to have narrow host ranges [5]. Studies of broad-host–range marine phages and metagenomic analyses of diverse environments suggest that such phages may be more common than previously thought in natural ecosystems [9–11]. Yet, little is known about the genetic determinants, evolutionary dynamics, trade-offs, and factors shaping host tropism that underlie the generalism of these phages in natural ecosystems.

Our previous research showed that juvenile oysters impacted by Pacific oyster mortality syndrome [12] are infected by diverse virulent genotypes within the *Vibrio crassostreae* species [13, 14]. These virulent strains are largely organized into distinct phylogenetic clades [15], or “patho-phylotypes”, and carry the virulence plasmid pGV, which encodes a T6SS responsible for cytotoxicity [16]. We also found this *V. crassostreae* population to be highly dynamic [13, 15], sparking our interest in the role of phages in shaping its abundance and diversity. To investigate, we collected marine phages and *V. crassostreae* hosts over a time series from an oyster farm and assessed their host range through cross-infection studies [15]. This analysis revealed a highly modular infection network, where phage adsorption is governed by specific matches between phage genus and vibrio clades. Following adsorption, intracellular defense systems further narrow the range of strains that are successfully infected and lysed.

One phage in our study, 6E351A, piqued our curiosity as the only isolate among 242 (0.4%) capable of infecting not only *V. crassostreae* strains outside any specific clade, but also strains from other *Vibrio* species [15]. Genome sequencing identified this phage as part of the Schizotequatrovirus family (previously known as giant vibriophages or T4 giant phages). These viruses, exhibiting a myovirus morphotype, are characterized by prolate capsids and double-stranded deoxyribonucleic acid (DNA) genomes of ~250 kbp, with a quarter of their coding regions showing homology to T4 phage genes [17]. The best-known member of this group, KVP40, is notable for its broad host range [18]. This finding suggests that Schizotequatroviruses may generally include broad-host–range phages. However, research on these phages is limited by the small number of isolated phages and hosts and the considerable genetic diversity within this family.

In this study, we established a collection of Schizotequatroviruses and their *V. crassostreae* hosts to investigate the fine-scale diversity and evolutionary dynamics within this family. Uncovering the genetic mechanisms and evolutionary processes that enable these phages to maintain a broad host range, along with understanding the potential trade-offs that may explain their low prevalence, will provide valuable insights into their role in shaping bacterial eco-evolutionary dynamics and inform their potential applications.

## Material and methods

### Isolation and identification of Schizotequatroviruses

Sampling was conducted from an oyster farm in the Bay of Brest (Pointe du Château, 48° 20′ 06.19′′ N, 4° 19′ 06.37′′ W) between 28 June and 15 September 2021, on Mondays, Wednesdays, and Fridays. Specific pathogen-free juvenile oysters [19] were deployed in consecutive batches, to monitor mortality linked to seawater temperature increases >16°C, a threshold for oyster mortalities. Samples were collected on each sampling day from living oysters within a batch showing <50% mortality. Hemolymph was collected from 90 living oysters, centrifuged (10 min, 17 000 g) and the supernatant filtered through a 0.2 μm filter and stored at 4°C until the phage isolation stage. Concurrently, 10 liters of seawater were collected and size fractionated as previously described [13] and viral particles were concentrated using 0.2 μm filtration and iron chloride flocculation, respectively, following standardized protocols [20]. Virus-flocculates were suspended in 10 ml 0.1 M EDTA, 0.2 M MgCl_2_, 0.2 M oxalate buffer at pH 6 and stored at 4°C until the phage isolation stage.

A total of 153 *V. crassostreae* isolates [15] were used as bait to isolate phages across 35 sampling dates. For each date, a mixture of 10 μl seawater viral concentrate (equivalent to 10 ml of seawater, concentrated 1000-fold) and 10 μl of oyster plasma derived from a pooled sample of 90 oysters was tested. Phage infections were identified by the formation of plaques using soft agar overlays on bacterial lawns. In a previous study [15], we collected up to six plaques per morphotype and found that they were mostly clonal. Therefore, we purified one phage per plaque morphotype and combination (host and date), resulting in a final collection of >1000 phages. Phages were re-isolated through up to three rounds of plaque purification to ensure purity. High-titer stocks (>10^9^ plaques forming units (PFU)/ml) were prepared via confluent lysis in agar overlays and stored at 4°C, with an additional aliquot stored at −80°C in the presence of 25% glycerol.

We initially prioritized sequencing phages isolating on strains within clades V1 to V5 and V8 (*n* = 861); however, none were identified as Schizotequatroviruses (data not shown). To address this gap, we designed three primer sets (Table S1) targeting conserved regions of Schizotequatrovirus genomes available in the NCBI RefSeq/GenBank database (Table S2). These primers were tested on crude lysates of phages isolated on strains outside clades (*n =* 183)*.* This targeted approach led to the detection of 17 phages, each producing polymerase chain reaction (PCR) products of the expected size with at least two primer pairs. Morphological analysis via electron microscopy and genome sequencing further confirmed their classification. Including phage 6E35.1, previously isolated in a time-series study [15], the collection of Schizotequatroviruses isolated from *V. crassostreae* now comprises 18 phages (Table S3).

### Electron microscopy

To characterize the morphology of the different phages, 20 μl of phage concentrates were adsorbed for 10 min to a formvar film on a carbon-coated 300 mesh copper grid (FF-300 Cu formvar square mesh Cu, delta microscopy). The adsorbed samples were negatively contrasted with 2% Uranyl acetate (EMS, Hatfield, PA, USA) before observation under a Jeol JEM-1400 Transmission Electron Microscope equipped with an Orious Gatan camera. Analyses were conducted by S. Le Panse at the platform MERIMAGE (Station Biologique, Roscoff, France).

### Phage genome sequencing and clustering

Phage suspensions (3 ml, >10^9^ PFU/ml) were concentrated using 1X PEG 8000 and 1 M NaCl, incubated overnight at 4°C, and centrifuged at 4500 rpm for 30 min at 4°C. The resulting pellet was resuspended in 300 μl SM buffer (100 mM NaCl, 8 mM MgSO₄·7H₂O, 50 mM Tris–Cl). Concentrated phages were then treated with DNAse (10 μl, 1000 units, Promega) and RNAse (2.5 μl, 3.5 mg/ml, Macherey–Nagel) at 37°C for 30 min. DNA extraction included protein lysis (0.02 M EDTA pH 8.0, 500 μg/ml proteinase K, 0.5% SDS) for 30 min at 55°C, phenol-chloroform extraction, and ethanol precipitation. DNA was visualized on a 0.7% agarose gel (50 V, overnight at 4°C) and quantified using Qubit.

Phage sequencing was performed at the Biomics platform of the Pasteur Institute (Paris, France). DNA was fragmented using Covaris (target size: 500 bp), and libraries were prepared with the TruSeq DNA PCR-Free High Library Prep Kit. Due to inefficiency in adaptor ligation, an amplification step of 14 cycles was added using Illumina P7 and P5 primers (IDT). Sequencing was carried out on a MiSeq Micro v2 flow cell (Illumina) with paired-end 2x150 cycles. Reads were trimmed using Trimmomatic v0.39 [21] and assembled de novo with SPAdes v3.15.2. Contaminant contigs were filtered using the UniVec Database, and the resulting one-contig phage genome was manually linearized.

Phages were clustered using VIRIDIC v1.0r3.6 with default settings [22]. Intergenomic similarities were determined through BLASTN pairwise comparisons, with virus classification into family (≥50% similarity), genus (≥70% similarity), and species (≥95% similarity) ranks following ICTV genome identity thresholds.

### Annotation of phage genome

Genome annotation was performed by Pharokka version 1.7.2 [23] with Phanotate version 1.5.0 employed for coding sequence (CDS) identification [24]. tRNAs genes were characterized using tRNAscan-SE 2.0 [25]. Additional functional information was obtained through eggNOG-mapper version 2.1.12 [26], querying all unique proteins of the dataset. Putative defense systems were identified based on genes returned by the HMM search subprocess [27] of DefenseFinder 1.3.0 [28] in two independent runs: one sensitive (--no-cut-ga option) and the other specific (default parameters). The same strategy was applied to antidefense systems, using DefenseFinder with the --antidefensefinder-only option [29]. Candidate receptor binding proteins (RBPs) were identified using PhageRBPdetection software version 3.0.0 [30] and one false positive among the eight hits was discarded due to an inconclusive AlphaFold-Multimer model [31]. Finally, all genes from the reference genome 6E351A were manually curated and classified into the 14 functional categories.

### Core genome phylogenies

Maximum likelihood core genome phylogenies of *V. crassostreae* and Schizotequatroviruses were produced by PanACoTA version 1.4.1 [32], with different parameters to account for the differential level of genetic divergence between the two populations (i.e. species level for hosts with an average nucleotide identity >95% for any pair of genomes, family level according to VIRIDIC for phages). The host phylogeny was inferred at the DNA level by IQ-TREE [33], employing 1000 ultrafast bootstraps and the GTR + F + I + G4 model of nucleotide substitution, which was assessed as the best fit according to the Bayesian Information Criterion (BIC). This phylogeny was derived from the concatenated multiple sequence alignment (MSA) of 2879 CDSs found in single copy in at least 99% of the genomes—this threshold was found to capture the majority of conserved single-copy gene families. Each marker set was defined as the CDSs of proteins sharing at least 80% amino-acid identity over at least 80% mutual length coverage, identified using the Linclust clustering algorithm from MMSeqs2 version 15-6f452 [34]. The MSA of each protein cluster was generated using MAFFT version 7.525 [35] and converted into a CDS alignment via amino acid to codon mapping. The root was known from a previous work in which *Vibrio gigantis* served as the outgroup [15]. In contrast, the phylogeny of phages was inferred at the protein level (1000 bootstraps, LG + G4 as the best model of amino-acid substitution) from the concatenated MSA of 264 proteins present in single copy in at least 90% of the genomes. Each marker set was defined by Linclust as proteins sharing at least 25% sequence identity over at least 80% of their length. The tree was rooted with F86 as the outgroup, which was consistent with the root identified by both the midpoint and the minimal ancestor deviation unsupervised methods [36].

### Phages pangenome, synteny plot, and fine-grained identification of core orthologs

Best-bidirectional hits (BBHs) were defined from an all-versus-all comparison of the 18 phage proteomes using MMseqs2 at maximal sensitivity (option -s 7.5), with an amino-acid identity cutoff of >20% and an alignment coverage of >50% of the length of the shortest protein. Groups of BBHs (BBH groups) were identified as connected components in the BBH graph, with 285 of the 547 BBH groups corresponding to core BBHs. 284 core BBH groups corresponded to single-copy BBHs whereas 1 core BBH group had a single copy in the F86 outgroup and 2 copies in the other 17 phages. For this group, we distinguished the two clusters of paralogs in the 17 phages using the OMA standalone orthology inference pipeline version 2.6.0 and only the paralogs found in the same OMA group as the unique protein of F86 were used in subsequent analyses of core genes. The pangenome graph was generated using the Python NetworkX version 3.2.1 package, with nodes representing BBH groups and edges indicating direct gene adjacency in at least one genome (weighted by the number of occurrences of the adjacency among the 18 genomes). The pangenome graph was visualized with Cytoscape version 3.10.0 [37] whereas the synteny plot was generated with the gggenomes R package version 0.9.12 (sequence identity scores of BBH pairs stored in the MMSeqs2 output were used to generate the links between proteins).

### Phylostratigraphy

To analyze events of gene loss, gain, and duplication, we leveraged the Hierarchical Orthologous Groups (HOGs) inferred during the previous run of the OMA standalone inference pipeline. As opposed to OMA groups which are inferred without a phylogeny and yields groups of orthologs with a unique member in each genome, a HOG is defined at each internal node of the recombination-free phylogeny and encompasses all descending paralogs and orthologs. HOGs are nested within HOGs of deeper levels in the phylogeny, up until the level of the “rootHOG”, namely the internal node of the phylogeny in which the gene family is inferred to have emerged/been gained. The 472 HOGs were mapped onto the nodes of the core genome recombination-free phylogeny using the ham function of pyHAM version 1.2.0 (Python HOGs Analysis Method) [38]. The phylostratigraphy plot was generated with the create_tree_profile function of pyHAM.

### Recombination events and recombination-free phylogeny

For each group of core BBHs, proteins were aligned using MAFFT with the options –pairwise and –maxiterate 1000 for high accuracy. The corresponding codon alignments were generated through amino acid to codon mapping. The CDS alignments were then concatenated based on the gene order in the reference strain 6E351A. A preliminary core genome phylogeny was inferred from the concatenated CDS alignment using the GTR + G4 model of nucleotide substitution with 1000 bootstraps, and it was rooted with F86 as the outgroup. To identify recombination events and create a recombination-free phylogeny, Gubbins version 3.3.0 [39] was applied to the same alignment, using the aforementioned phylogeny as a starting tree and employing IQ-TREE with the GTR + G4 model of nucleotide substitution for tree construction. The topologies of recombination-unaware and -free phylogenies were compared using the Phylo.io online tool [40].

### Alignment-wide nonsynonymous to synonymous substitution

The MSA of proteins from each group of core orthologous genes was trimmed with Trimal version 1.4.1 to retain only sites with <20% gaps [41]. The corresponding trimmed CDS alignments were generated using amino acid to codon mapping. For each trimmed CDS alignment, we applied Hyphy version 2.5.63 [42] to estimate a single alignment-wide dN/dS, using the recombination-free phylogeny as the guide tree and utilizing the recommended MG94CUSTOMCF3X4 model of codon substitution along with the GTR model of nucleotide substitution (command-line: hyphy acd Universal $CDS_MSA MG94CUSTOMCF3X4 Global 012345 $RECOMBFREE_TREE Estimate).

### Phages–bacteria interactions analyses

The heatmap of the cross-infection matrix was produced with the ComplexHeatmap R package version 2.15.4 [43]. The specialization index of a phage is represented by its Paired Difference Index (PDI), as introduced by Poisot *et al.* in the context of bacteria–phage ecology [44]. This index measures specificity as differential exploitation of bacterial isolates, by contrasting the highest infection score achieved on a host (PFU_1_) with those achieved on the other hosts (PFU_2_ to PFU_n_). The PDI for a given phage is therefore calculated as: 


$$\text{PDI}_{\text{phage}}= \frac{\sum_{i=2}^{Nb\ hosts}\left({log}10\left( PF{U}_1\right)-{log}10\left( PF{U}_i\right)\right)}{Nb\ hosts-1}. $$

Similarly, the susceptibility score of a host was given as: 


$$\text{Sscore}_{\text{host}} =1 - \text{PDI}_{\text{host}}=1- \frac{\sum_{i=2}^{Nb\ phages}\left({log}10\left( PF{U}_1\right)-{log}10\left( PF{U}_i\right)\right)}{Nb\ phages-1.} $$

where PFU_1_ is the highest infection score recorded by a phage on the assessed host. Null PFU scores were treated as non-assigned values and PDI values were computed with the species level function of the R bipartite package. Hosts were classified into three distinct classes based on the susceptibility score distribution: resistant (score of 0), slightly susceptible (score >0 but <0.5), susceptible (score ≥0.5). The hypothesis that the distribution of either polymorphism (patristic distances), specificity to oysters, or specificity to the French West Coast of hosts with respect to their respective six closest strains differs across the three susceptibility classes was tested using the non-parametric Kruskal–Wallis test. Subsequent post-hoc pairwise tests were conducted using the non-parametric Mann–Whitney U test.

### Phylogenetic profiling of OmpK and LamB

The OmpK and LamB families were identified as single linkage protein clusters (connected components in the sequence similarity graph) in the MMSeqs2 all-vs-all analysis of host proteomes, filtered for pairwise alignments yielding a least 25% identity and 70% mutual coverage. Identical proteins within each family were deduplicated at 100% sequence identity using the -c 1 option of CD-HIT version 4.8.1 [45]. For each family, all unique protein variants were aligned with MAFFT, and a maximum-likelihood phylogeny was inferred from the MSA with IQ-TREE, utilizing the -m TEST option for finding the best model of amino-acid substitution (1000 bootstraps). Each tree was rooted using the midpoint method. Finally, for each protein family, a presence/absence matrix of variants across the host population was created. The matrix was visualized with the ComplexHeatmap R package [43] clustered according to the gene tree (rows) and the host phylogeny (columns).

### Structural analysis and prediction of the OmpK and LamB RBPs

To identify putative RBPs for OmpK and LamB, AlphaFold-Multimer [46] was utilized to perform pairwise structural predictions of complexes between OmpK and each of the seven candidate RBPs from phage 6E351A identified by PhageRBPdetection software, as well as LamB and the phage K566 orthologs of these seven candidate RBPs. The models of the OmpK-RBP complexes were unreliable, displaying non-physiologically relevant interaction interfaces and/or high inter-chain predicted aligned error (PAE) scores ≥30 Å; therefore, no conclusions could be drawn from their analysis. However, several predicted LamB–RBP complexes were of sufficient quality (inter-chain PAE scores <10 Å) that they could reflect a physiological receptor-RBP arrangement. To further narrow the list of candidates, the sequences of these RBPs were examined to identify K566/K567-specific variations that mapped to the predicted LamB–RBP interaction interface. Only one candidate, VPK566_0420/VPK567_0422, exhibited a difference in this region that could account for changes in receptor specificity, therefore this was assigned as the LamB RBP for phages K566 and K567.

Structures of *V. crassostreae* OmpK and LamB, as well as the seven candidate RBPs from phage 6E351A and K566, were predicted using AlphaFold2 [47] and AlphaFold-Multimer as implemented in ColabFold [48], or AlphaFold3 [49] as appropriate. Identification of sequence-level conservation of protein domains was performed using CD-Search [50]. Structural similarity searches against the Protein Data Bank (PDB) were performed using the Foldseek [51] and DALI [52] servers. Structural alignments were generated using the PDB Pairwise Structure Alignment tool [53]. Calculation of electrostatic surface potential was performed using the Adaptive Poisson–Boltzmann Solver (APBS) service [54]. Visualization of predicted structures was performed using ChimeraX [55]. MSAs were generated using the default parameters of Muscle (version 5.1) as implemented in Geneious Prime 2025.0.2.

### Measurement of phage traits

Host range assays were carried out using an electronic multichannel pipette by spotting 10-fold serial dilutions onto host lawns. Plates were incubated overnight at RT and plaque formation was observed after 24 h.

Phage adsorption experiments were conducted as described previously [15]. Phages were mixed with exponentially growing cells (OD 0.3; 10^7^ CFU/ml) at a multiplicity of infection (MOI) of 0.01 and incubated at room temperature without agitation. At set intervals, 1 ml of culture was transferred into a tube with 100 μl of chloroform, centrifuged at 14 000 rpm for 5 min. The supernatant was serially diluted and drop-spotted onto a sensitive host lawn to quantify remaining free phages.

Burst size and latency period were estimated using a protocol provided by the S. Moineau laboratory (Université Laval, Québec, Canada). Phages were mixed with exponentially growing cells (OD 0.3; 10^7^ CFU/ml) at an MOI of 0.01 and incubated at room temperature until maximum adsorption. Bacteria were then washed twice, and the pellet was resuspended in a 1/100 dilution of fresh media. Every 10 min, an aliquot of the culture was collected and plated (either pure or diluted) onto a host lawn. After overnight incubation at room temperature, plaques were counted after 24 h. Burst size was estimated as the average number of plaques at the first plateau divided by the average plaque count before the initial increase in plaque numbers (infection center). The latent period was defined as the time elapsed from initial phage adsorption to the first observed host cell lysis, determined as the last time point before PFU counts began to increase, plus the adsorption duration.

### Proteomic analysis of phage particles

Phage suspensions (10^10^ PFU) were precipitated using PEG as previously described, and the pellet was resuspended in 500 μl RIPA buffer (0.1% SDS, 1% Triton, 1 mM EDTA, 10 mM Tris–HCl, pH 8). Protein concentration was measured using the Biorad Protein Assay according to the manufacturer’s instructions, and samples were stored in 100 μl aliquots at −80°C. A total of 20 μg of protein was digested by trypsin and analyzed by tandem mass spectrometry. Peptides were mapped to the 6E351A phage proteome, and protein identities were assigned using the Protein Prophet algorithm [56] and validated with Scaffold (version Scaffold_5.3.3, Proteome Software Inc., Portland, OR). Peptide identifications were validated with a probability >80%, and protein identifications with a probability >90% with at least one identified peptide [57]. Proteins with similar peptides that could not be differentiated were grouped. Protein quantification and identification are listed in Table S4. Analyses were conducted by D. Faubert and M. Boulos, at the Proteomics Discovery Platform at the Montreal Clinical Research Institute, Canada.

### Detection of modified bases in phage deoxyribonucleic acid

DNA from phage 6E351A was purified using the Monarch® RNA Cleanup Kit (NEB), and 0.5 μg of DNA was hydrolyzed with a nucleoside digestion mix (NEB). The resulting nucleoside solution was analyzed by HPLC and mass spectrometry (LC/MS) [58]. The HPLC-UV trace of 6E351A was aligned and compared to DNA digests from phage Lambda and the Bacillus phage PBP1, which contains dPreQ0 in its virion DNA.

### Digital droplet polymerase chain reaction

A total of 2 ml of viral concentrates from seawater or plasma-derived viruses were used for DNA extraction (2 sources, 35 dates, totaling 70 DNA samples). Phages were concentrated by adding 1X PEG 8000 and 1 M NaCl, followed by overnight incubation at 4°C and centrifugation at 4500 rpm for 30 min at 4°C. The resulting pellet was resuspended in 300 μl of SM buffer, DNA was then extracted following previously described protocols, resuspended in 100 μl and quantified using a Nanodrop, with an average concentration of 100 ng/μl.

Droplet digital PCR (ddPCR) reactions consisted of 20 μl mixture per well containing 10 μl of ddPCR Evagreen Supermix, 600 nM of primers (Table S1) and 5 μl of DNA diluted 1/4. The ddPCR reactions were incorporated into droplets using the QX100 Droplet Generator (Bio-Rad). Nucleic acids were amplified with the following cycling conditions: 5 min at 95°C, 40 cycles of 30 s at 95°C and 60°C for 60 s, and a final droplet cure step of 5 min at 4°C then 5 min at 90°C using a Bio-Rad’s C1000 Touch Thermal Cycler. Droplets were read and analyzed using Bio-Rad QX600 system and QuantaSoft software (version 1.7.4.0917) in “absolute quantification” mode. Only wells containing ≥10 000 droplets were accepted for further analysis.

### Molecular microbiology

Bacterial strains and plasmids used in this study are listed in Tables S5 and S6. *V. crassostreae* isolates were grown on marine agar (MA) or broth (MB) at room temperature with gentle agitation, whereas *Escherichia coli* strains were cultured in Lysogeny Broth (LB) at 37°C with shaking. Where necessary, chloramphenicol (Cm; 5 or 25 μg/ml for *V. crassostreae* and *E. coli*, respectively), thymidine (0.3 mM), and diaminopimelate (0.3 mM) were added to the media. The P_BAD_ promoter was induced with 0.2% L-arabinose and repressed with 1% D-glucose.

Cloning was performed with Gibson Assembly (New England Biolabs, NEB), with all constructs confirmed by sequencing. Gene inactivation was achieved using two methods: (i) Gene deletion was performed by cloning flanking regions into the pSW7848T suicide plasmid [59], allowing selection of mutants via P_BAD_ -*ccdB* regulated integration and elimination steps, confirmed by PCR [60]; (ii) an internal region of the target gene was cloned into pSW23T [61], and single crossover integration was used for inactivation. For complementation, target genes or a *gfp* control were cloned under the P_LAC_ promoter in a P_MRB_ based vector [62] and introduced into mutants by conjugation.

## Results

### Isolation of Schizotequatroviruses

**Figure 1:**
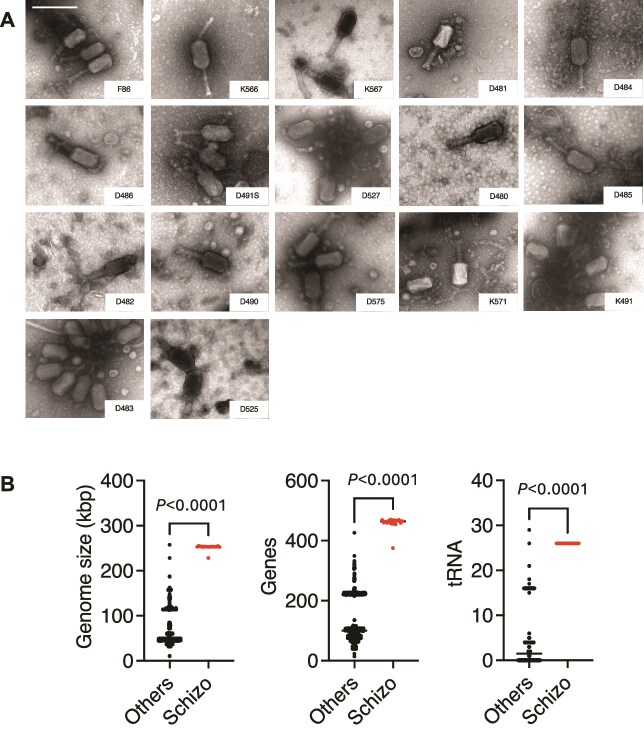
Morphological and genomic distinctions of newly isolated Schizotequatroviruses. (A) Transmission electron micrograph of 17 newly isolated Schizotequatroviruses produced using diverse *V. crassostreae* as host. Images are representative of the observation of dozens of particles. Scale bar: 200 nm. (B) Characteristics of the 18 Schizotequatroviruses relative to the other 1000 phages. *P* values are for unpaired t-tests.

To expand our search for Schizotequatroviruses, we conducted a new time-series sampling at the same oyster farm (Brest, spanning 35 dates from 1 July to 15 September 2021). This endeavor resulted in the generation of archives of virus concentrates from both seawater and oyster plasma. Utilizing our prior collection of *V. crassostreae* strains [15], we successfully isolated and sequenced 1044 viruses. We identified 17 new phages (1.7%) classified by the Virus Intergenomic Distance Calculator (VIRIDIC, [22]) within the Schizotequatrovirus family (Table S7). Upon further examination through transmission electron microscopy (TEM), a striking morphological resemblance was observed between the 17 newly isolated Schizotequatroviruses (Fig. 1A) and the previously characterized Schizotequatroviruses 6E351A [15] all of which were isolated from *V. crassostreae*. These phages were characterized by a prolate head ~125 nm in size and a contractile tail measuring ~110 nm. Genome sequencing unveiled an average genome length of 252 203 bp (+/− 5983 bp) with 349–444 predicted coding sequences (CDSs) and precisely 26 tRNAs (Fig. 1B, Table S3).

### Large core genome of the Schizotequatrovirus

To characterize the variability of Schizotequatrovirus gene repertoires, we first identified the groups of orthologs (bidirectional best hits, BBHs) between the 18 phage genomes (6E351A plus the 17 new phages). On this basis, the pan-genome of the Schizotequatrovirus consisted of 543 gene families, of which 285 are families of core genes, i.e. with a member in all 18 genomes. Almost half (48.6%) of the core genes lacked a known function. Most families are either present in >80% of the genomes (76.6%) or in <20% (17.5%; Fig. S1), comparable to what is observed in bacterial genomes [63]. Characterization of the gene functions, followed by manual curation in phage 6E351A, resulted in 178 BBH groups that could be classed in 14 distinct functional categories (Table S8). One set of core genes encodes various components of viral particles [64], including the head, tail, long tail fibers, collar, and whiskers and a proteomic analysis of phage particles confirmed the function of the structural genes (Tables S4 and S8). We identified no less than seven tail fibers (ranging from 474 to 1223 aa in length) using the PhageRBPdetection software that could be RBPs [30]. Other persistent genes govern processes crucial for viral transcription, replication, procapsid assembly, genome packaging, tail assembly and host cell lysis to release the phage progeny (Table S8). Finally, all genomes of the Schizotequatrovirus contain 26 tRNAs (Fig. 1B and Table S3).

### Known repertoire of defenses and counter-defenses

The phage pan-genome includes numerous defense and counter-defense systems, aligning with the capacity of large-genome phages to accumulate such functions (Table S8). The accessory genome of the Schizotequatrovirus includes 2 BBH groups of Viperins and a component of the PD-T4-9 defense system. Surprisingly, even though the repertoire of anti-defense systems usually evolves fast, we found several that are part of the core genome, e.g. the homologs of the NARP2 protein which circumvents NAD+ depletion of the Acb1 anti-CBASS protein [65] and of the AcrIIA7 anti-CRISPR protein. We also identified a core 7-deazaguanine derivative biosynthesis gene cluster [66], which may play a role in restriction modification (R-M) system evasion. Four enzymes—FolE, QueD, QueE, and QueC—are predicted to synthesize the precursor 7-cyano-7-deazaguanine (*preQ0*) from GTP. DpdA likely mediates the incorporation of *preQ0* into DNA as *dpreQ0*, a modified guanine base previously observed in Schizotequatrovirus phage nt-1 with 0.1% guanine modification [67]. In phages containing QueF, an additional modification to 7-aminomethyl-7-deazaguanine (*preQ1*) occurs, modifying the DpdA substrate to preQ1. The presence of QueF in the 6E351A genome within this gene cluster suggests a dG modification to *dPreQ1* in its DNA. Mass spectrometric analysis of extracted DNA (Fig. S2) confirmed a 15% dG modification, with an observed mass of 295 Da, consistent with *dPreQ1*. Of note, we detected the FolE component of the 7-deazaguanine derivative biosynthesis gene cluster as well as the Acb1 Anti-CBASS and NARP2 NAD+ anti-depletion proteins in viral particles (Table S8), hinting that the phage can deploy these antidefense proteins to immediately defend itself upon infection.

We examined the alignment between phage counter-defense genes and host defense systems in 21 host strains previously identified as susceptible to phage 6E351A [15]. The 7-deazaguanine derivative biosynthesis gene cluster corresponded to the most frequently observed host defense systems, particularly R-M types I and II (Table S9). Additionally, the Acb1, NARP2, and AcrIIA7 genes aligned with host defense systems such as CBASS_I, CBASS_II, and CAS Class 1-Subtype-I-F, which were each detected in at least one of the analyzed strains. With a total of 58 known defense systems identified across these susceptible strains, Schizotequatroviruses may harbor yet-undiscovered antidefense mechanisms capable of countering these diverse host defenses.

### Impact of recombination in Schizotequatroviruses

**Figure 2:**
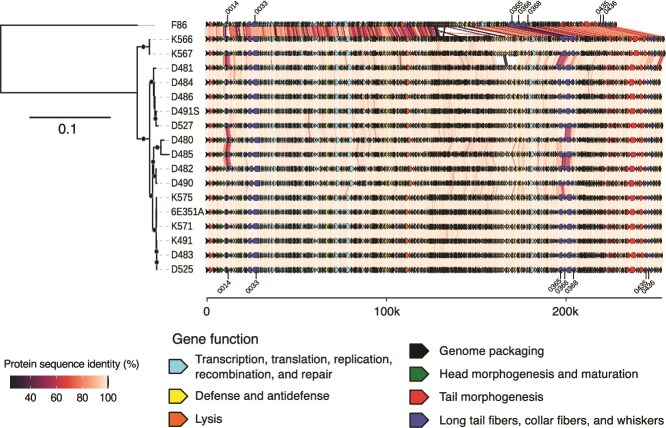
Maximum likelihood phylogeny and genetic organization of Schizotequatroviruses. Black dots indicate branches with over 90% bootstrap support. Genetic organization visualization was generated with the gggenomes R package. Protein-coding genes are colored according to their functional category. Genes of uncharacterized function are represented in gray. Pairwise conservation of genetic organization is represented by links connecting BBHs and colors denote amino-acid sequence identity. The seven groups of BBHs encoding candidate RBPs are labeled in phages 6E351A and D525.

The previous results suggest a large conservation of gene composition among Schizotequatroviruses, with a few events of gene gains and losses. To better understand the evolution of these phages, we built a phylogenetic tree of a restricted set of single-copy gene families present in at least 17 of the 18 genomes, (Fig. 2). The phylogeny of Schizotequatroviruses unambiguously flags phage F86 as the outgroup of the clade. The clade is further divided into two subclades: one composed of 15 phages, and the other containing the two phages K566 and K567. The genetic organization is remarkably conserved across the entire set of genomes, even when including the divergent F86 outgroup. Yet, the network of gene adjacencies reveals a few highly variable regions. These regions are enriched in uncharacterized proteins and in genes involved in defense/anti-defense systems, in line with their rapid turnover and their classification as accessory genes (Fig. S1).

When excluding the F86 outgroup, most BBHs appear nearly identical between phages (Fig. 2). The exceptions concern mostly loci encoding putative RBPs (Fig. 2), which suggests that cell adsorption is a fast-evolving trait in these phages. We wondered whether divergence in these loci resulted from homologous recombination events or rapid evolution by point mutations. We used Gubbins [39] to identify the events of homologous recombination in the recombination-corrected phylogenetic tree. Among the 202 predicted events of recombination 13 were on internal and 16 on terminal branches of the phylogeny (Fig. S3). In total, 236 of the 285 core genes have evidence of recombination during the diversification of the phage. Yet, some loci have recombined more than the others (Fig. S4). The two main recombination hotspots in Schizotequatroviruses correspond to the 125–130 kb and 195–205 kb regions in 6E351A (Fig. 2), which encode proteins of uncharacterized functions and long tail fiber RBPs, respectively. These two regions include core loci inferred to have undergone 9 recombination events during the diversification of the phage.

To assess if high sequence divergence is caused by episodes of positive selection, we estimated the gene-wide strength of selection acting against amino-acid substitution in core genes with Hyphy [42]. This was obtained from the ratio of nonsynonymous to synonymous substitution (dN/dS) substitution rates. A dN/dS ratio <1 suggests purifying selection, and a ratio higher than 1 suggests positive selection. All persistent genes had a dN/dS <0.4 (Fig. S4). Even though this does not exclude positive selection at a few sites in the proteins, it does reveal a strong imprint of purifying selection. Some RBP BBHs share <60% identity in the clade of the 17 closely-related phages when the minimal pairwise identity in the vast majority of core BBH groups is above 90% (Table S8). To understand the reason of these differences, we compared the mean pairwise identity between BBHs within the most divergent core BBH groups (defined as a mean pairwise identity <95% in the clade of 17 phages) and the number of inferred recombination events and the dN/dS ratio (Fig. S5). The results show a significant association of divergence with recombination and no association with the dN/dS ratio. This further suggests that divergence in core genes of Schizotequatroviruses is associated with recombination processes and not caused by selection for rapid accumulation of amino-acid substitutions from ancestral alleles.

The branch lengths and topology of the phylogeny inferred at the DNA level from all persistent genes present in the 18 genomes is substantially different than the recombination-free phylogeny inferred by Gubbins (Fig. S6), and only the latter is congruent with the robust phylogeny inferred at the protein-level from conservative BBH groups (Fig. 2). This affects the position of the phages K566 and K567 because recombination accounts for most nucleotide substitutions between their core genes and those of the 15 members of the sister subclade (Fig. S6). When recombination signals are removed by Gubbins, the two subclades appear to have diverged at the same rate of nucleotide substitution from their last common ancestor. The inferred history of gene gains, duplications and losses (Fig. S7) identified another founding event of the shift of K566 and K567: the replacement of an ancestral anti-defense island (VP6E351A_0314 to _0326) by another one (VPK566_0302 to _0314 in phage K566) providing new functions, e.g. a glycosyltransferase and genes involved in Lipid A biosynthesis (VPK566_0309 and _0313 in Table S8). Hence, the emergence of the sub-clade K566 and K567 is associated with events of recombination and acquisition of several genes. Together, these results suggest that recombination played a critical role in establishing polymorphism within Schizotequatroviruses in genes that have a key role in phage-bacteria initial interactions.

### Variations in host range

**Figure 3:**
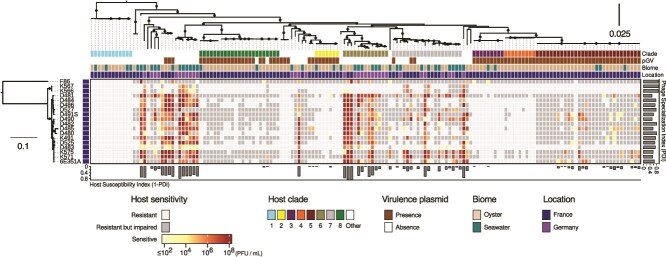
Schizotequatrovirus—*Vibrio crassostreae* quantitative interaction matrix. The central heat map displays the results of 2826 cross-infection assays (18 phages × 157 hosts). Rows represent phages and columns represent hosts, both ordered based on their core genome phylogeny. The scale of the phage (bacterial) phylogeny corresponds to the estimated average amino-acid (nucleotide) substitution per site. The four colored strips aligned with the host phylogeny indicate: (i) the eight bacterial clades [15], (ii) presence of the virulence pGV plasmid [13], (iii) environment of isolation (biome), and (iv) sampling location. The color of each cell on the heatmap indicates the efficiency of phage infection (in PFU)/ml). Light gray cells indicate bacterial resistance to the phage. Dark gray cells indicate that the host is impaired when challenged by high phage titers, but no production of viable phages is observed (a sign of adsorption, but no productive infection).

To finely quantify the host range of the 18 Schizotequatroviruses isolated from *V. crassostreae*, we first measured the titer of each phage on 157 *V. crassostreae* strains, including 124 isolates from the same location (Brest, France) and 33 isolates from a different location (Sylt, Germany; Fig. 3). In sensitive hosts, the phage titers ranged from <10^2^ to 10^8^ PFU/ml. The host strain was classified as “resistant but impaired” if we observed a clearing zone but no production of viable phages and as “resistant” if no clearing zone nor plaque was observed.

We observed variability in host range and efficiency of infection across the Schizotequatroviruses population (Fig. 3). We assessed the differential exploitation of bacterial isolates of each phage by computing the PDI, a metric introduced to study specificity in the context of bacteria–phage ecology [44, 68]. The higher a PDI is for a phage, the greater is the contrast between the highest infection score it can achieve on a host and the infection scores achieved on the other hosts. The F86 outgroup had a PDI of 0.89, K566/567 were found to be the most specialized with PDIs of 0.90 and 0.91, and the subclade of 15 phages had the broadest host range with an average PDI of 0.68 (Fig. 3).

To broaden the diversity of population tested for host range, we compared the titer of phages 6E351A (representative of the subclade of 15 phages) and K567 (representative of the K566/567 subclade) on four additional *Vibrio* species (*Vibrio cyclitrophicus*, *V. tasmaniensis, Vibrio splendidus*, and *Vibrio breoganii*) encompassing isolates from Brest or Plum Island (MA, USA) [69]. Strains permitting phage reproduction were identified in all four species independently of their location (Fig. S8A). Hence, infection patterns of phages observed between strains of *V. crassostreae* was reproduced for strains of the four other species, namely that K567 infected fewer strains of the same species than 6E351A (Fig. S8B).

We analyzed the Schizotequatroviruses–*V. crassostreae* interaction matrix from the perspective of the infected host. We computed a susceptibility score for each host, given as 1 - PDI_host_, where the PDI of a host contrasts the highest infection score achieved by a phage on the host with the scores achieved by the other phages (Fig. 3). The distribution of susceptibility scores clearly delineated three classes of hosts: resistant (score of 0), slightly susceptible (score >0 but <0.5) and susceptible (score of at least 0.5; Fig. S9A). All strains from clade V1 were resistant to the 18 phages (Fig. 3), suggesting that the diversification of this clade was accompanied by the loss of Schizotequatroviruses receptor(s). Strains from the “patho-phylotypes” (V2 to V5 and V8) carrying the virulence plasmid pGV [13, 16] were either resistant or slightly susceptible (Fig. 3) although a good proportion of these strains were classified as resistant but impaired, indicating successful adsorption by the phages. The highest proportion of sensitive strains belonged to clades V6 and V7 or did not fall into any specific clades from [15] (Fig. 3).

The Schizotequatroviruses (all sampled in Brest) are more likely to infect clades of *V. crassostreae* which (i) are characterized by a substantial polymorphism in core genes (Fig. S9B), (ii) are not specific to Brest (Fig. S9D), and (iii) are not found exclusively in oysters (Fig. S9C). Strains from populations in locations without intensive oyster farming, such as Plum Island (USA) and Sylt (Germany), including *V. breoganii*, an algae specialist [70] were also susceptible to Schizotequatroviruses (Fig. S8). This provides an interesting ecological insight, namely that the generalist Schizotequatroviruses tend to target clades of generalist vibrios rather than clades of quasi-clonal patho-phylotype vibrios well adapted to a specific niche and known to be infected by specialist phages [15].

### OmpK and LamB are receptors for the Schizotequatroviruses

The previous results suggest that diversification of the phage core genes occurs primarily at the functions involved in the first steps of infection. To better understand the patterns of *V. crassostreae* - Schizotequatroviruses interactions, we thus sought to identify the attachment site of the phages on *V. crassostreae* and the cognate protein in the phage.

**Figure 4:**
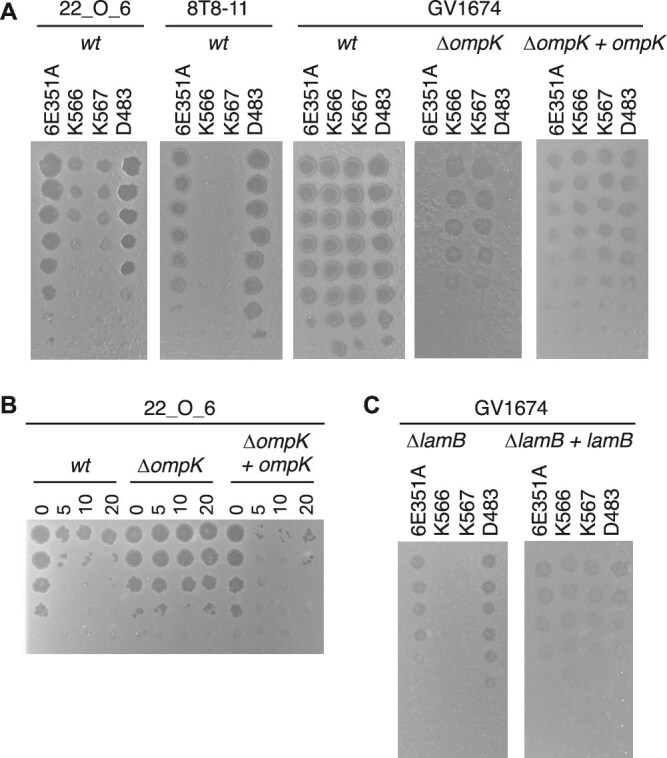
Role of OmpK and LamB as a receptor for Schizotequatroviruses. (A) Killing assays were performed by spotting 10-fold serial dilutions of each indicated phage onto the wild-type strain (wt), an *∆ompK* mutant derivative, and the *∆ompK* strain complemented with a plasmid constitutively expressing *ompK*. The tested strains are the original strains used for phage isolation: 22_O_6 for 6E351A, 8 T8–11 for D483, and GV1674 for K566 and K567. (B) Adsorption assay for phage 6E351A on the wt strain 22_O_6 and its *∆ompK* mutant. A fixed concentration of phages (MOI 0.01) was allowed to adsorb to each strain for the indicated times, after which unattached (free) phages were serially diluted and plated with the original host, 22_O_6. A decrease in infectious particles indicates successful phage adsorption. (C) Killing assays performed on the *∆lamB* mutant of GV1674 and its complemented derivative.

**Figure 5:**
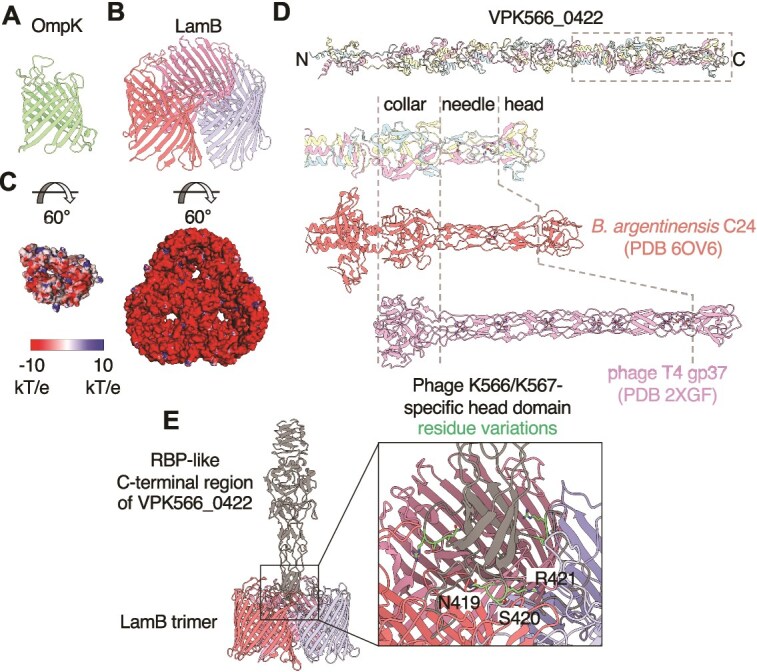
Phages K566 and K567 encode a putative RBP that may recognize LamB. (A) structure of *V. crassostreae* OmpK predicted by AlphaFold2 (AF2). (B) Structure of *V. crassostreae* LamB predicted as a homotrimer by AlphaFold-Multimer. (C) Electrostatic representation of the extracellular-facing surface of OmpK (left) and LamB (right) calculated using APBS; contoured from −10 kT/e to 10 kT/e. (D) (Top) Structure of VPK566_0422 predicted as a homotrimer by AlphaFold3. The amino (N) and carboxy (C) termini are indicated. The dashed box indicates the RBP-like region. (Bottom) Comparison of the RBP-like region of VPK566_0422 to *Bizionia argentinensis* phage tip protein C24 (PDB 6OV6) and phage T4 long tail fiber tip protein gp37 (PDB 2XGF), drawn to scale. The shared collar, needle, and head domains of these proteins are indicated. Magnesium ions coordinated by the needle domains are depicted as spheres. (E) (Left) Complex of LamB and VPK566_0422 (residues 295–476; gray), predicted by AlphaFold-Multimer. (Right) Close-up view of the head domain interaction with LamB. The phage K566- and K567-specific sequence variations are highlighted. The addition of an electropositive arginine residue may facilitate interactions with the more electronegative extracellular-facing surface of LamB.

The phage KVP40 receptor was previously identified as the outer membrane protein K (OmpK), a protein of unknown function constitutively expressed on the cell surface [71]. Therefore, we investigated whether our collection of Schizotequatroviruses also requires OmpK to infect *V. crassostreae*. For 16 out of 18 phages the deletion of *ompK* in the host resulted in a resistant phenotype whereas the constitutive expression of *ompK* from a plasmid was sufficient to restore the infectivity of the phages (Fig. 4A, Fig. S10). Adsorption assays further supported the hypothesis that OmpK is the receptor for phage 6E351A (Fig. 4B). Consistent with these observations, the only clade of the *V. crassostreae* which is fully resistant to the Schizotequatrovirus (clade V1 in Fig. 3) lacks the *ompK* gene (Fig. S11). AlphaFold2 predicted that OmpK adopts a 12-stranded β-barrel structure (Fig. 5A and Fig. S12A), with strong similarity to the monomeric *E. coli* nucleoside transporter Tsx (PDB 1TLW, rmsd 2.05 Å over 215 Cα).

We found that phages K566 and K567 do not require OmpK for infection (Fig. 4A). To identify their receptor(s), we generated spontaneous mutants from their original strain (GV1674) or its Δ*ompK* derivative. A total of 40 mutants were selected for genome sequencing, revealing 1 to 3 single nucleotide polymorphisms in the phage-resistant isolates (Table S10). Among the 10 genes identified with nonsense mutations, frameshifts, or nonsynonymous mutations, two genes were affected, encoding the maltoporin LamB (10/40 strains) and its regulator MalT (20/40 strains). LamB has been previously reported as the main receptor for phages λ [72], T4-K10 [73], and T-even-like phage Tula [74]. To confirm that LamB serves as a receptor for phages K566 and K567, we constructed a *lamB* deletion mutant in the original host strain (GV1674). The two phages failed to infect the single mutant Δ*lamB*, and the constitutive expression of *lamB* from a plasmid was sufficient to restore the infectivity of the phages (Fig. 4C). LamB was predicted to adopt a trimeric 18-stranded β-barrel (Fig. 5B and Fig. S12B) with strong similarity to a *Salmonella typhimurium* maltoporin (PDB 1MPR, rmsd 2.4 Å over 348 Cα).

 We conclude than OmpK and LamB are receptors of this family of phages in *V. crassostreae*. To understand the potential impact of the change in receptors in phage-bacteria interactions, we searched to identify their cognate phage proteins. LamB is significantly more electronegative than OmpK (Fig. 5C) and using AlphaFold-Multimer we found that VPK566_0422 is the best candidate for interaction with the LamB RBP in phages K566/K567 (no candidate obtained for OmpK; Fig. S13). This protein exhibits weak similarity to the receptor binding tip protein C24 of a prophage in *Bizionia argentinensis* [75] (Fig. 5D) and phage T4 protein gp37, which recognizes the trimeric outer membrane β-barrel OmpC in *E. coli* [76]. The unique ability of phages K566 and K567 to use LamB for adsorption was probably the result of a single event of homologous recombination identified at VPK566_0422 in the last common ancestor of K566 and K567 (Fig. S3). The VPK566_0422 specific residues in phage K566/K567 are optimally positioned to participate in bacterial receptor interactions (Fig. 5E, Fig. S14).

### Environmental abundance and life cycle dynamics of Schizotequatroviruses

**Figure 6:**
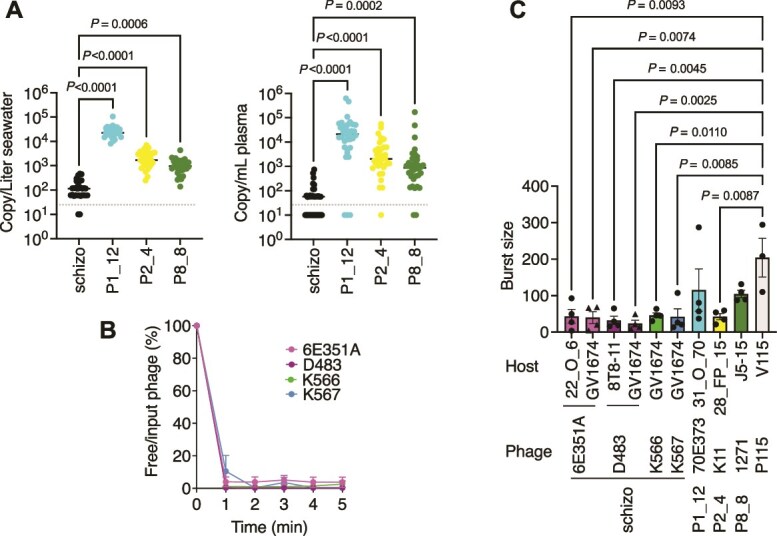
Environmental abundance and life cycle dynamics of Schizotequatroviruses. (A) Absolute abundance of Schizotequatroviruses and other phage genera infecting *V. crassostreae* (respectively, clades V1, V2, and V8) in seawater (left) and oyster plasma (right). Genome copies per liter of seawater or ml of oyster plasma were quantified using ddPCR. DNA samples were prepared from virome sources collected >35 dates during the 2021 time series. The dashed line represents the detection limit. A Friedman test on RM one-way ANOVA results indicates that Schizotequatroviruses have significantly lower abundance compared to other phages in both oyster plasma and seawater. (B) Adsorption rates were calculated as the ratio of free phages to input phages at each time point. Data represent three independent experiments. (C) Burst sizes of the indicated phages were determined using one-step growth curves on original or alternative hosts. Bars represent the mean ± standard error of the mean (SEM) from four independent experiments. Ordinary one-way ANOVA with Tukey’s multiple comparisons test shows a significant difference between all Schizotequatroviruses and P115, regardless of the host used to estimate burst size.

Our results show that Schizotequatroviruses have broad host range and can adapt to match different receptors in bacteria. Yet, they are rare in our samples (1.7% of the collection). Under culture conditions, broad host range phages were shown to have slower growth rates and be outcompeted by narrower host range phages with higher virulence [5]. To assess if such types of trade-offs could explain our observations, we employed a culture-independent technique, ddPCR, to estimate the absolute abundance of phages in seawater and oyster plasma over the time series. When detected, Schizotequatrovirus genomes ranged from 57 to 470 copies per liter of seawater and 57 to 748 copies per milliliter of oyster plasma (Fig. S15). The abundance of Schizotequatroviruses was significantly lower than that of other phage genera, which specialize in phylo-pathotypes (Fig. 6A). We conclude that despite their broad host range, extracellular Schizotequatroviruses’ relative abundance remains low in this environment.

Host range shows a strong negative correlation with reproduction rate, whereas decay exhibits a positive correlation with reproduction rate, based on available data [77]. The reproduction rate of a phage is primarily determined by three factors: adsorption rate (the speed at which a phage irreversibly binds to a host cell, initiating the infection process), latent period (the time from host cell infection to the release of new viruses), and burst size (the number of viral offspring produced per infected host cell). We therefore determined these parameters to identify which could be at a trade-off with host range and explain the low frequency of the Schizotequatrovirus.

Phage adsorption assays were performed by mixing phages with exponentially growing cells at a MOI of 0.01, followed by monitoring the loss of free phages over time. We previously observed that phage 6E351A rapidly adsorbed to its original host strain, 22_O_6 (Figs. 4b). This rapid adsorption rate (within 1 min) was further confirmed with strain GV1674 using four representative Schizotequatroviruses (6E351A, D483, K566, and K567), regardless of whether their receptor was OmpK or LamB (Fig. 6B).

The latent period and burst size were determined using a one-step growth curve with an MOI of 0.01. The latent period was estimated to be 30–40 min (Fig. S16), aligning with the bacterial generation time. The burst size averaged 38 ± 27 particles per cell, with no significant differences observed across phage–host combinations (Fig. 6C, Fig. S16). The burst size of Schizotequatroviruses was similar to that of narrower-host–range phages from genera P1_12, P2_4, and P8_8, which infect *V. crassostreae* from clades V1, V2, and V8, respectively. However, phage P115, which is highly specific to a strain of *Vibrio chagasii* [78, 79] exhibited a significantly higher burst size (Fig. 6C). Collectively, these findings indicate that the lower environmental abundance of Schizotequatroviruses compared to specialist phages targeting clades of *V. crassostreae* is not attributable to differences in burst size, as estimated *in vitro.* Although these *in vitro* results fail to account for their relatively low abundance, they highlight the limitations of laboratory experiments in replicating the complexity of natural ecosystems. Future studies employing more “eco-realistic” approaches will be essential to uncover the trade-offs between their broad host range and replication dynamics.

## Discussion

Our study reveals the genetic mechanisms and evolutionary processes that enable Schizotequatroviruses to sustain a generalist lifestyle. Schizotequatroviruses are highly conserved exhibiting a large set of core genes and conserved genetic architecture. But they also show the ability to diversify quickly regarding some specific traits. This is exemplified by their acquisition and rapid turnover of multiple RBPs, enabling adaptation to receptor mutations, as well as antidefense systems. Most Schizotequatroviruses, including the F86 outgroup and the distant KVP40 phage, use OmpK for adsorption. However, a subclade of two phages requires instead LamB for adsorption, indicating a receptor switch from OmpK to LamB. This subclade diversified through the acquisition of a novel antidefense island and a recombination event in a collar fiber, with the new allele inferred as the LamB RBP. Together, our results highlight recombination as a key mechanism supporting broad host range, particularly through the exchange of tail fibers, allowing phages to overcome selective pressures from host defenses and competition with more virulent specialists. Recombination between virulent phages requires co-infection but was recently shown to be frequent [80].

The relative scarcity of Schizotequatroviruses in the environment suggests they face fitness costs that offset the benefits of a broad host range [5, 81]. *In vitro* experiments showed their rapid adsorption rates can be both an advantage and a liability—allowing quick host access but also risking attachment to non-viable targets, reducing fitness [77]. Although slow replication rates are observed, this trait is not unique to generalist phages, as some specialists also exhibit similar rates. Laboratory assessments, however, often overlook ecological factors like phage dispersion and persistence, host density, and spatial structure. Future studies should explore reproduction dynamics under ecologically relevant conditions and leverage single-cell analyses to unravel the sustainability paradox of these broad-host–range but environmentally rare phages.

We observed that Schizotequatroviruses tend to target clades of generalist hosts whose members are found among different geographic locations and biomes rather than the patho-phylotypes specifically adapted to oysters. These generalist vibrios, often avirulent in oysters, likely enter the oyster environment passively via filter-feeding and thrive in open seawater [13]. In contrast, we showed that patho-phylotypes—those that bloomed in diseased oysters—were predominantly infected by specialist phages, rather than by the generalist Schizotequatrovirus 6E351 [15]. Oyster farming practices may actively select for narrow bacterial clades and their corresponding specialist phages, which are abundant in this environment. Investigating Schizotequatroviruses outside epidemic events may reveal a higher environmental prevalence and clarify their ecological role.

Schizotequatroviruses’ large genomes offer notable advantages, including the ability to encode diverse RBPs and antidefense mechanisms, enabling broad host range. These genomes also support regulatory complexity, optimizing infection based on host physiological states [82]. However, large genomes might come with trade-offs: they require more resources and time for replication, resulting in slower replication rates [83–85], and may depend on sophisticated host machinery, limiting the range of potential hosts. Additionally, the increased genetic content presents more targets for host defenses like CRISPR-Cas systems. Exploring Schizotequatroviruses alongside other large-genome viruses could shed light on the interplay between genome size, host range, and evolutionary viability [86].

Considering long-term applications, such as preventing vibriosis in aquaculture, previous studies demonstrated the efficacy of KVP40 in reducing fish larval mortality [87], despite eventual resistance development [88–90]. For juvenile oysters, our findings suggest that a single Schizotequatrovirus may not adequately cover the genetic diversity of *V. crassostreae* pathogens. A cocktail of specialist phages might provide more robust protection. Nonetheless, elucidating the molecular mechanisms underlying Schizotequatroviruses’ broad host range—particularly antidefense systems—holds promise for engineering phages tailored to specifically target patho-phylotypes sustainably and effectively.

## Supplementary Material

Supplementary_Figures_wraf063

All_tables_sup_wraf063

## Data Availability

The sequenced Schizotequatrovirus genomes have been deposited in the NCBI database, with accession numbers listed in Table S3.

## Materials availability

The Schizotequatrovirus and their *Vibrio* hosts have been deposited in the following public collection: https://roscoff-culture-collection.org/ with accession numbers listed in Tables S3 and S5. All mutants and plasmids are available upon request to FLR.

## References

[1] Breitbart M, Bonnain C, Malki K. et al. Phage puppet masters of the marine microbial realm. *Nat Microbiol* 2018;3:754–66. 10.1038/s41564-018-0166-y 29867096

[2] Ofir G, Sorek R. Contemporary phage biology: from classic models to new insights. *Cell* 2018;172:1260–70. 10.1016/j.cell.2017.10.045 29522746

[3] Salmond GP, Fineran PC. A century of the phage: past, present and future. *Nat Rev Microbiol* 2015;13:777–86. 10.1038/nrmicro3564 26548913

[4] Strathdee SA, Hatfull GF, Mutalik VK. et al. Phage therapy: from biological mechanisms to future directions. *Cell* 2023;186:17–31. 10.1016/j.cell.2022.11.017 36608652 PMC9827498

[5] de Jonge PA, Nobrega FL, Brouns SJJ. et al. Molecular and evolutionary determinants of bacteriophage host range. *Trends Microbiol* 2019;27:51–63. 30181062 10.1016/j.tim.2018.08.006

[6] Holtappels D, Alfenas-Zerbini P, Koskella B. Drivers and consequences of bacteriophage host range. *FEMS Microbiol Rev* 2023;47:1–10. 10.1093/femsre/fuad038 37422441

[7] Georjon H, Bernheim A. The highly diverse antiphage defence systems of bacteria. *Nat Rev Microbiol* 2023;21:686–700. 10.1038/s41579-023-00934-x 37460672

[8] Koskella B, Hernandez CA, Wheatley RM. Understanding the impacts of bacteriophage viruses: from laboratory evolution to natural ecosystems. *Annu Rev Virol* 2022;9:57–78. 35584889 10.1146/annurev-virology-091919-075914

[9] Dekel-Bird NP, Sabehi G, Mosevitzky B. et al. Host-dependent differences in abundance, composition and host range of cyanophages from the Red Sea. *Environ Microbiol* 2015;17:1286–99. 25041521 10.1111/1462-2920.12569

[10] Kauffman KM, Hussain FA, Yang J. et al. A major lineage of non-tailed dsDNA viruses as unrecognized killers of marine bacteria. *Nature* 2018;554:118–22. 10.1038/nature25474 29364876

[11] Munson-McGee JH, Peng S, Dewerff S. et al. A virus or more in (nearly) every cell: ubiquitous networks of virus-host interactions in extreme environments. *ISME J* 2018;12:1706–14. 10.1038/s41396-018-0071-7 29467398 PMC6018696

[12] de Lorgeril J, Lucasson A, Petton B. et al. Immune-suppression by OsHV-1 viral infection causes fatal bacteraemia in Pacific oysters. *Nat Commun* 2018;9:4215. 10.1038/s41467-018-06659-3 30310074 PMC6182001

[13] Bruto M, James A, Petton B. et al. *Vibrio crassostreae,* a benign oyster colonizer turned into a pathogen after plasmid acquisition. *ISME J.* 2017;11:1043–52. 10.1038/ismej.2016.162 27922600 PMC5364345

[14] Lemire A, Goudenège D, Versigny T. et al. Populations, not clones, are the unit of vibrio pathogenesis in naturally infected oysters. *The ISME journal* 2015;9:1523–31. 25489729 10.1038/ismej.2014.233 PMC4478691

[15] Piel D, Bruto M, Labreuche Y. et al. Phage–host coevolution in natural populations. *Nat Microbiol* 2022;7:1075–86. 10.1038/s41564-022-01157-1 35760840

[16] Piel D, Bruto M, James A. et al. Selection of *vibrio crassostreae* relies on a plasmid expressing a type 6 secretion system cytotoxic for host immune cells. *Environ Microbiol* 2020;22:4198–211. 31390475 10.1111/1462-2920.14776

[17] Miller ES, Heidelberg JF, Eisen JA. et al. Complete genome sequence of the broad-host-range vibriophage KVP40: comparative genomics of a T4-related bacteriophage. *J Bacteriol* 2003;185:5220–33. 10.1128/JB.185.17.5220-5233.2003 12923095 PMC180978

[18] Matsuzaki S, Tanaka S, Koga T. et al. A broad-host-range vibriophage, KVP40, isolated from sea water. *Microbiol Immunol* 1992;36:93–7. 1584076 10.1111/j.1348-0421.1992.tb01645.x

[19] Le Roux F, Wegner KM, Polz MF. Oysters and vibrios as a model for disease dynamics in wild animals. *Trends Microbiol* 2016;24:568–80. 10.1016/j.tim.2016.03.006 27038736

[20] Kauffman KM, Polz MF. Streamlining standard bacteriophage methods for higher throughput. *MethodsX* 2018;5:159–72. 10.1016/j.mex.2018.01.007 30622914 PMC6318102

[21] Bolger AM, Lohse M, Usadel B. Trimmomatic: a flexible trimmer for Illumina sequence data. *Bioinformatics* 2014;30:2114–20. 24695404 10.1093/bioinformatics/btu170 PMC4103590

[22] Moraru C, Varsani A, Kropinski AM. VIRIDIC-a novel tool to calculate the intergenomic similarities of prokaryote-infecting viruses. *Viruses* 2020;12:1268. 10.3390/v12111268 33172115 PMC7694805

[23] Bouras G, Nepal R, Houtak G. et al. Pharokka: a fast scalable bacteriophage annotation tool. *Bioinformatics* 2023;39:776. 10.1093/bioinformatics/btac776 PMC9805569 36453861

[24] McNair K, Zhou C, Dinsdale EA. et al. PHANOTATE: a novel approach to gene identification in phage genomes. *Bioinformatics* 2019;35:4537–42. 10.1093/bioinformatics/btz265 31329826 PMC6853651

[25] Chan PP, Lin BY, Mak AJ. et al. tRNAscan-SE 2.0: improved detection and functional classification of transfer RNA genes. *Nucleic Acids Res* 2021;49:9077–96. 10.1093/nar/gkab688 34417604 PMC8450103

[26] Cantalapiedra CP, Hernandez-Plaza A, Letunic I. et al. eggNOG-mapper v2: functional annotation, orthology assignments, and domain prediction at the metagenomic scale. *Mol Biol Evol* 2021;38:5825–9. 10.1093/molbev/msab293 34597405 PMC8662613

[27] Eddy SR . Accelerated profile HMM searches. *PLoS Comput Biol* 2011;7:e1002195. 10.1371/journal.pcbi.1002195 22039361 PMC3197634

[28] Tesson F, Herve A, Mordret E. et al. Systematic and quantitative view of the antiviral arsenal of prokaryotes. *Nat Commun* 2022;13:2561. 10.1038/s41467-022-30269-9 35538097 PMC9090908

[29] Tesson F, Huiting E, Wei L. et al. Exploring the diversity of anti-defense systems across prokaryotes, phages, and mobile genetic elements. Nucleic Acids Res 2025;53:gkae1171. 10.1093/nar/gkae1171. PMC11724313 39657785

[30] Boeckaerts D, Stock M, De Baets B. et al. Identification of phage receptor-binding protein sequences with hidden Markov models and an extreme gradient boosting classifier. *viruses* 2022;14:1329. 10.3390/v14061329 PMC9230537 35746800

[31] Evans R, O’Neill M, Pritzel A. et al. Protein complex prediction with AlphaFold-Multimer. bioRxiv. 2022:2021.10.04.463034.

[32] Perrin A, Rocha EPC. PanACoTA: a modular tool for massive microbial comparative genomics. *NAR Genom Bioinform* 2021;3:lqaa106. 33575648 10.1093/nargab/lqaa106 PMC7803007

[33] Minh BQ, Schmidt HA, Chernomor O. et al. IQ-TREE 2: new models and efficient methods for phylogenetic inference in the genomic era. *Mol Biol Evol* 2020;37:1530–4. 32011700 10.1093/molbev/msaa015 PMC7182206

[34] Steinegger M, Soding J. Clustering huge protein sequence sets in linear time. *Nat Commun* 2018;9:2542. 10.1038/s41467-018-04964-5 29959318 PMC6026198

[35] Katoh K, Standley DM. MAFFT multiple sequence alignment software version 7: improvements in performance and usability. *Mol Biol Evol* 2013;30:772–80. 10.1093/molbev/mst010 23329690 PMC3603318

[36] Tria FDK, Landan G, Dagan T. Phylogenetic rooting using minimal ancestor deviation. *Nat Ecol Evol* 2017;1:193. 10.1038/s41559-017-0193 29388565

[37] Shannon P, Markiel A, Ozier O. et al. Cytoscape: a software environment for integrated models of biomolecular interaction networks. *Genome Res* 2003;13:2498–504. 14597658 10.1101/gr.1239303 PMC403769

[38] Train CM, Pignatelli M, Altenhoff A. et al. iHam and pyHam: visualizing and processing hierarchical orthologous groups. *Bioinformatics* 2019;35:2504–6. 10.1093/bioinformatics/bty994 30508066 PMC6612847

[39] Croucher NJ, Page AJ, Connor TR. et al. Rapid phylogenetic analysis of large samples of recombinant bacterial whole genome sequences using Gubbins. *Nucleic Acids Res* 2015;43:e15. 10.1093/nar/gku1196 25414349 PMC4330336

[40] Robinson O, Dylus D, Dessimoz C. Phylo.Io: interactive viewing and comparison of large phylogenetic trees on the web. *Mol Biol Evol* 2016;33:2163–6. 27189561 10.1093/molbev/msw080 PMC4948708

[41] Capella-Gutierrez S, Silla-Martinez JM, Gabaldon T. trimAl: a tool for automated alignment trimming in large-scale phylogenetic analyses. *Bioinformatics* 2009;25:1972–3. 10.1093/bioinformatics/btp348 19505945 PMC2712344

[42] Kosakovsky Pond SL, Poon AFY, Velazquez R. et al. HyPhy 2.5-a customizable platform for evolutionary hypothesis testing using phylogenies. *Mol Biol Evol* 2020;37:295–9. 10.1093/molbev/msz197 31504749 PMC8204705

[43] Gu Z . Complex heatmap visualization. *iMeta* 2022;1:e43. 10.1002/imt2.43 38868715 PMC10989952

[44] Poisot T, Lepennetier G, Martinez E. et al. Resource availability affects the structure of a natural bacteria-bacteriophage community. *Biol Lett* 2011;7:201–4. 10.1098/rsbl.2010.0774 20961886 PMC3061169

[45] Li W, Godzik A. Cd-hit: a fast program for clustering and comparing large sets of protein or nucleotide sequences. *Bioinformatics* 2006;22:1658–9. 16731699 10.1093/bioinformatics/btl158

[46] Evans R, O’Neill M, Pritzel A. et al. Protein complex prediction with alphaFold-multimer. bioRxiv 2021.10.04.463034; 10.1101/2021.10.04.463034

[47] Jumper J, Evans R, Pritzel A. et al. Highly accurate protein structure prediction with alphaFold. *Nature* 2021;596:583–9. 34265844 10.1038/s41586-021-03819-2 PMC8371605

[48] Mirdita M, Schutze K, Moriwaki Y. et al. ColabFold: making protein folding accessible to all. *Nat Methods* 2022;19:679–82. 35637307 10.1038/s41592-022-01488-1 PMC9184281

[49] Abramson J, Adler J, Dunger J. et al. Accurate structure prediction of biomolecular interactions with AlphaFold 3. *Nature* 2024;630:493–500. 10.1038/s41586-024-07487-w 38718835 PMC11168924

[50] Chen Y, Nie F, Xie SQ. et al. Efficient assembly of nanopore reads via highly accurate and intact error correction. *Nat Commun* 2021;12:60. 33397900 10.1038/s41467-020-20236-7 PMC7782737

[51] van Kempen M, Kim SS, Tumescheit C. et al. Fast and accurate protein structure search with Foldseek. *Nat Biotechnol* 2024;42:243–6. 37156916 10.1038/s41587-023-01773-0 PMC10869269

[52] Holm L, Laiho A, Toronen P. et al. DALI shines a light on remote homologs: one hundred discoveries. *Protein Sci* 2023;32:e4519. 10.1002/pro.4519 36419248 PMC9793968

[53] Bittrich S, Segura J, Duarte JM. et al. RCSB protein data bank: exploring protein 3D similarities via comprehensive structural alignments. *Bioinformatics* 2024;40:370. 10.1093/bioinformatics/btae370 PMC11212067 38870521

[54] Jurrus E, Engel D, Star K. et al. Improvements to the APBS biomolecular solvation software suite. *Protein Sci* 2018;27:112–28. 10.1002/pro.3280 28836357 PMC5734301

[55] Meng EC, Goddard TD, Pettersen EF. et al. UCSF ChimeraX: tools for structure building and analysis. *Protein Sci* 2023;32:e4792. 10.1002/pro.4792 37774136 PMC10588335

[56] Nesvizhskii AI, Keller A, Kolker E. et al. A statistical model for identifying proteins by tandem mass spectrometry. *Anal Chem* 2003;75:4646–58. 14632076 10.1021/ac0341261

[57] Keller A, Nesvizhskii AI, Kolker E. et al. Empirical statistical model to estimate the accuracy of peptide identifications made by MS/MS and database search. *Anal Chem* 2002;74:5383–92. 10.1021/ac025747h 12403597

[58] Lee YJ, Weigele PR. Detection of modified bases in bacteriophage genomic DNA. *Methods Mol Biol* 2021;2198:53–66. 32822022 10.1007/978-1-0716-0876-0_5

[59] Val ME, Skovgaard O, Ducos-Galand M. et al. Genome engineering in *Vibrio cholerae*: a feasible approach to address biological issues. *PLoS Genet* 2012;8:e1002472. 10.1371/journal.pgen.1002472 22253612 PMC3257285

[60] Le Roux F, Binesse J, Saulnier D. et al. Construction of a *Vibrio splendidus* mutant lacking the metalloprotease gene *vsm* by use of a novel counterselectable suicide vector. *Appl Environ Microbiol* 2007;73:777–84. 17122399 10.1128/AEM.02147-06 PMC1800747

[61] Demarre G, Guerout AM, Matsumoto-Mashimo C. et al. A new family of mobilizable suicide plasmids based on broad host range R388 plasmid (IncW) and RP4 plasmid (IncPalpha) conjugative machineries and their cognate *Escherichia coli* host strains. *Res Microbiol* 2005;156:245–55. 15748991 10.1016/j.resmic.2004.09.007

[62] Le Roux F, Davis BM, Waldor MK. Conserved small RNAs govern replication and incompatibility of a diverse new plasmid family from marine bacteria. *Nucleic Acids Res* 2011;39:1004–13. 20923782 10.1093/nar/gkq852 PMC3035462

[63] Francis AR, Tanaka MM. Evolution of variation in presence and absence of genes in bacterial pathways. *BMC Evol Biol* 2012;12:55. 22520826 10.1186/1471-2148-12-55 PMC3514204

[64] Yap ML, Rossmann MG. Structure and function of bacteriophage T4. *Future Microbiol* 2014;9:1319–27. 25517898 10.2217/fmb.14.91 PMC4275845

[65] Osterman I, Samra H, Rousset F. et al. Phages reconstitute NAD(+) to counter bacterial immunity. *Nature* 2024;634:1160–7. 10.1038/s41586-024-07986-w 39322677

[66] de Crecy-Lagard V, Hutinet G, Cediel-Becerra JDD. et al. Biosynthesis and function of 7-deazaguanine derivatives in bacteria and phages. *Microbiol Mol Biol Rev* 2024;88:e0019923. 10.1128/mmbr.00199-23 38421302 PMC10966956

[67] Hutinet G, Kot W, Cui L. et al. 7-Deazaguanine modifications protect phage DNA from host restriction systems. *Nat Commun* 2019;10:5442. 31784519 10.1038/s41467-019-13384-y PMC6884629

[68] Poisot T, Canard E, Mouquet N. et al. A comparative study of ecological specialization estimators. *Methods Ecol Evol* 2012;3:537–44. 10.1111/j.2041-210X.2011.00174.x

[69] Bruto M, Labreuche Y, James A. et al. Ancestral gene acquisition as the key to virulence potential in environmental *Vibrio* populations. *ISME J* 2018;12:2954–66. 10.1038/s41396-018-0245-3 30072747 PMC6246604

[70] Corzett CH, Elsherbini J, Chien DM. et al. Evolution of a vegetarian *Vibrio*: metabolic specialization of *Vibrio breoganii* to macroalgal substrates. *J Bacteriol* 2018;200:e00020–18. 10.1128/JB.00020-18 29632094 PMC6040190

[71] Inoue T, Matsuzaki S, Tanaka S. A 26-kDa outer membrane protein, OmpK, common to *Vibrio* species is the receptor for a broad-host-range vibriophage, KVP40. *FEMS Microbiol Lett* 1995;125:101–5. 10.1111/j.1574-6968.1995.tb07342.x 7867914

[72] Randall-Hazelbauer L, Schwartz M. Isolation of the bacteriophage lambda receptor from *Escherichia coli*. *J Bacteriol* 1973;116:1436–46. 10.1128/jb.116.3.1436-1446.1973 4201774 PMC246503

[73] Roa M . Interaction of bacteriophage K10 with its receptor, the LamB protein of *Escherichia coli*. *J Bacteriol* 1979;140:680–6. 10.1128/jb.140.2.680-686.1979 387746 PMC216697

[74] Wandersman C, Schwartz M. Protein La and the LamB protein can replace each other in the constitution of an active receptor for the same coliphage. *Proc Natl Acad Sci USA* 1978;75:5636–9. 10.1073/pnas.75.11.5636 364487 PMC393022

[75] Pellizza L, Lopez JL, Vazquez S. et al. Structure of the putative long tail fiber receptor-binding tip of a novel temperate bacteriophage from the antarctic bacterium *Bizionia argentinensis* JUB59. *J Struct Biol* 2020;212:107595. 10.1016/j.jsb.2020.107595 32736071

[76] Bartual SG, Otero JM, Garcia-Doval C. et al. Structure of the bacteriophage T4 long tail fiber receptor-binding tip. *Proc Natl Acad Sci USA* 2010;107:20287–92. 10.1073/pnas.1011218107 21041684 PMC2996694

[77] Keen EC . Tradeoffs in bacteriophage life histories. *Bacteriophage* 2014;4:e28365. 10.4161/bact.28365 24616839 PMC3942329

[78] Barcia-Cruz R, Goudenege D, Moura de Sousa JA. et al. Phage-inducible chromosomal minimalist islands (PICMIs), a novel family of small marine satellites of virulent phages. *Nat Commun* 2024;15:664. 10.1038/s41467-024-44965-1 PMC10803314 38253718

[79] Cahier K, Piel D, Barcia-Cruz R. et al. Environmental vibrio phage-bacteria interaction networks reflect the genetic structure of host populations. *Environ Microbiol* 2023;25:1424–38. 36876921 10.1111/1462-2920.16366

[80] Kauffman KM, Chang WK, Brown JM. et al. Resolving the structure of phage-bacteria interactions in the context of natural diversity. *Nat Commun* 2022;13:372. 10.1038/s41467-021-27583-z 35042853 PMC8766483

[81] Bono LM, Draghi JA, Turner PE. Evolvability costs of niche expansion. *Trends Genet* 2020;36:14–23. 10.1016/j.tig.2019.10.003 31699305

[82] Al-Shayeb B, Sachdeva R, Chen LX. et al. Clades of huge phages from across earth's ecosystems. *Nature* 2020;578:425–31. 10.1038/s41586-020-2007-4 32051592 PMC7162821

[83] da Silva JD, Melo LDR, Santos SB. et al. Genomic and proteomic characterization of vB_SauM-UFV_DC4, a novel *Staphylococcus* jumbo phage. *Appl Microbiol Biotechnol* 2023;107:7231–50. 37741937 10.1007/s00253-023-12743-6 PMC10638138

[84] Hu M, Xing B, Yang M. et al. Characterization of a novel genus of jumbo phages and their application in wastewater treatment. *iScience* 2023;26:106947. 10.1016/j.isci.2023.106947 37324530 PMC10265529

[85] Sharma R, Pielstick BA, Bell KA. et al. A novel, highly related jumbo family of bacteriophages that were isolated against *Erwinia*. *Front Microbiol* 2019;10:1533. 31428059 10.3389/fmicb.2019.01533 PMC6690015

[86] Edwards KF, Steward GF, Schvarcz CR. Making sense of virus size and the tradeoffs shaping viral fitness. *Ecol Lett* 2021;24:363–73. 10.1111/ele.13630 33146939

[87] Rorbo N, Ronneseth A, Kalatzis PG. et al. Exploring the effect of phage therapy in preventing *vibrio anguillarum* infections in cod and turbot larvae. *Antibiotics (Basel)* 2018;7:42. 10.3390/antibiotics7020042 29772736 PMC6023099

[88] Castillo D, Rorbo N, Jorgensen J. et al. Phage defense mechanisms and their genomic and phenotypic implications in the fish pathogen *Vibrio anguillarum*. *FEMS Microbiol Ecol* 2019;95:10.1093/femsec/fiz004. 10.1093/femsec/fiz004 30624625

[89] Tan D, Dahl A, Middelboe M. Vibriophages differentially influence biofilm formation by *vibrio anguillarum* strains. *Appl Environ Microbiol* 2015;81:4489–97. 10.1128/AEM.00518-15 25911474 PMC4475874

[90] Tan D, Svenningsen SL, Middelboe M. Quorum sensing determines the choice of antiphage defense strategy in *Vibrio anguillarum*. *MBio* 2015;6:e00627. 10.1128/mBio.00627-15 26081633 PMC4471561

